# VOICE-ACTIVATED DIGITAL ASSISTANTS: PERCEPTIONS FROM NOVICE USERS WITH LONG-TERM MOBILITY DISABILITY

**DOI:** 10.1093/geroni/igz038.2791

**Published:** 2019-11-08

**Authors:** Lyndsie M Koon, Kenneth Blocker, Wendy Rogers

**Affiliations:** 1 University of Illinois, Urbana-Champaign, Illinois, United States; 2 University of Illinois at Urbana Champaign, Champaign, Illinois, United States; 3 University of Illinois, Champaign, Illinois, United States

## Abstract

Voice-activated digital assistants (e.g., Amazon Echo, Google Home) are an emerging technology that have great potential to provide support for adults aging with a long-term mobility disability. Digital assistant technologies allow the user to perform a variety of everyday tasks and activities through voice interactions. Such tasks may include environmental control (e.g., turning on/off lights, voice-activated temperature control); supporting self-health management (e.g., providing medication reminders, encouraging physical activity engagement); and fostering opportunities for social engagement (e.g., messaging/calling others, playing games remotely). This presentation will focus on the perceived facilitators and barriers to digital assistant use in the home among adults aging with mobility disabilities. The findings provide design guidelines and insight for intervention implementation for the use of these technologies for the target population.

